# Major evolutionary transitions in individuality between humans and AI

**DOI:** 10.1098/rstb.2021.0408

**Published:** 2023-03-13

**Authors:** Paul B. Rainey

**Affiliations:** ^1^ Department of Microbial Population Biology, Max Planck Institute for Evolutionary Biology, 24306 Plön, Germany; ^2^ Laboratoire Biophysique et Évolution, CBI, ESPCI Paris, Université PSL, CNRS, 75005 Paris, France

**Keywords:** Darwinian individuality, ecological scaffolding, heredity, interactions, life cycles, symbiosis

## Abstract

That humans might undergo future evolutionary transitions in individuality (ETIs) seems fanciful. However, drawing upon recent thinking concerning the origins of properties that underpin ETIs, I argue that certain ETIs are imminently realizable. Central to my argument is recognition that heritable variance in fitness at higher levels of organization can be externally imposed (scaffolded) by specific ecological structures and cultural practices. While ETIs to eusociality seem highly improbable, ETIs involving symbioses between humans and artificial intelligence (AI) can be readily envisaged. A necessary requirement is that fitness-affecting interactions between humans and AI devices are inherited by offspring. The Mendelian nature of human reproduction ensures that offspring resemble parents. Reproduction of AI devices requires nothing more than transference of algorithms from parental AI devices to devices that are assigned to offspring. This simple copying, combined with societal structures that require humans to carry AI devices, ensures heritable variance in fitness at the level of both interacting partners. Selection at the collective level will drive alignment of replicative fates and increase co-dependency, thus alleviating need for continual imposition of externally imposed scaffolds. I conclude by drawing attention to the immediacy of such transitions and express concern over possibilities for malevolent manipulation.

This article is part of the theme issue ‘Human socio-cultural evolution in light of evolutionary transitions’.

## 1. Introduction

Human societies are complex, ever changing and uniquely different from all other species. While uniqueness is not evident in genetic distinctiveness from other species, it is apparent in components of human evolution, including elements of life history, but especially language, cognition and cultural transmission [1].

Recognizing that evolution of biological complexity has been marked by a small number of events of major significance, involving, among other things, increases in complexity and changes in the way that information is stored and transmitted, Maynard Smith & Szathmáry [2] argued that humans are the product of a major evolutionary transition. Given rapid changes in technology and ensuing effects on culture [3]—both of which stand to further affect the storage and transmission of information [4]—it is pertinent and timely to consider whether humans are currently undergoing, or are poised to undergo, a further major evolutionary transition.

In this article, I aim to contribute to this discussion but wish to do so from a perspective that places emphasis on major evolutionary transitions in *individuality* (ETIs). In placing emphasis on individuality, a distinction is made between major evolutionary transitions that are marked by changes in the way information is stored and transmitted, and those that additionally involve the emergence of a new, clearly defined, higher-level unit of selection. Viewed from this standpoint, and as elaborated below, it is challenging, but not impossible (see, for example, [5–8]), to argue that humans are the product of a major ETI. What interests me, though, is the possibility that humans are currently undergoing [9], or might undergo, at some future time, an ETI of paradigmatic form. I will argue that fraternal transitions to eusociality, in the sense of beehives or termite mounds, is improbable [10]. However, I contend that egalitarian transitions between humans and artificial intelligence (AI) devices are likely, might emerge in the absence of specific intent, and could be readily engineered via societal-level control of behaviour.

## 2. Darwinian individuality and life's hierarchical structure

Darwinian populations are composed of individuals that differ one to another, replicate and leave offspring [11,12]. The rules of Mendelian inheritance ensure that offspring resemble parental types. Provided some component of variation affects reproductive success, then Darwinian individuals—those entities being endowed with Darwinian properties—participate in the process of evolution by natural selection. The outcome is adaption—the evolution of features that determine the fit between organism and environment.

Individuality manifests at multiple organization levels [13,14]. A set of Matryoshka dolls presents a suitable metaphor. Take for example a eusocial insect colony as evident in the hives of honeybees. Colonies are Darwinian: colonies vary one to another, colonies leave colony-level offspring and offspring colonies, being derived from the germline of a single queen, resemble parental colonies. Colonies thus manifest heritable variance in fitness and as a consequence participate in the process of evolution by natural selection as colonies in their own right. In turn, colonies are composed of multicellular bees that also manifest heritable variance in fitness. The same is true of the cells of which multicellular bees are composed. Inside cells there exist organelles (mitochondria) replete with remnants of ancestral eubacterial DNA. The nucleus contains chromosomes, and chromosomes are composed of genes. In principle it is possible to extend the set of nested replicators back in time to the first autocatalytic chemical reactions that emerged from matter, but doing so is fraught with challenges arising from lack of extant examples.

The set of nested replicators defines life's hierarchical structure [13]. Because each level manifests some degree of heritable variance in fitness, each level participates in the process of evolution by natural selection. This stated, functional integrity of higher-level collectives depends on function of lower-level entities and thus the evolutionary potential of lower-level entities is typically constrained through slavish adherence to development programmes [13]. Nonetheless, evidence of the potential of selection to affect the fate of lower-level particles, for example, cells in the case of multicellular organism, can be seen when mutations arise that cause cells to forgo faithful observance of instructions to cease division. Cells carrying such mutations proliferate unchecked giving rise to cancerous tumours that stand to impede function of multicellular entities. Selection operating at multiple levels typically generates conflict: what is in the interest of the higher level is not necessarily in the short-term interest of the lower level [15].

The nesting of lower-level particles within higher-level self-replicating entities begs questions as to the origin of life's hierarchical structure. A wealth of evidence points to life having evolved via a series of major ETIs in which lower-level entities became subsumed within higher-level collectives [2]: chromosomes evolved from genes, the eukaryotic cell arose from the merger of two once free-living prokaryotes, multicellular organisms evolved from unicellular types, and eusocial societies from multicellular organisms. A pertinent distinction concerns whether transitions are fraternal, that is, they arise from a single entity, as in the evolution of multicellular life from unicellular types, or are egalitarian, that is, they arise from the coming together of separate entities, as in evolution of the eukaryotic cell [16].

Understanding the causes of ETIs requires knowledge of how collectives composed of lower-level entities acquire Darwinian properties sufficient to allow collectives to participate directly in the process of evolution by natural selection [17–21]. Just how these properties emerge is an issue of fundamental importance [22,23]. There has been a tendency to sweep the problem aside, assuming that what exists at the lower level is somehow magically transitioned to the higher level. But as elaborated elsewhere, discreteness (and thus variation), replication and heredity are derived traits and their origin requires evolutionary explanation [22–25].

In some instances, genes performing some function at the lower level might be fortuitously co-opted to effect a higher-level function. For example, in snowflake yeast, genes controlling apoptosis at the single-cell level are integral to fragmentation (and thus reproduction) of the arms of snowflake collectives [26]. In other instances, it is not obvious that co-option is possible. In these situations, explaining the origin of Darwinian properties is challenging—especially so given the need to avoid explanations that pre-suppose the presence of Darwinian properties at the level at which they emerge [23,27]. For instance, consider the need to explain the evolution of collective-level reproduction: reproduction is a complex process and thus it seems reasonable to invoke natural selection (at the collective level) as its primary cause; however, to do so is to invoke the trait requiring explanation as the cause of its own evolution.

Faced with the challenge of explaining how a Darwinian process emerges among entities that are themselves non-Darwinian, it becomes necessary to seek new solutions. Possibilities arise from recognition that certain environmental conditions can exogenously impose Darwinian properties on nascent collectives, thus causing lower-level particles to participate unwittingly in a selective process that unfolds at the level of collectives [28]. The concept, termed ‘ecological scaffolding’, serves to direct attention to environments where scaffolding processes might naturally unfold, and to the construction of highly contrived scenarios for top-down engineering of ETIs [23]. The latter has special relevance for thinking about the evolution of symbioses between humans and AI that might even evolve to the point where the two interacting partners replicate as one.

## 3. Ecological scaffolding

The hypothesis of ecological scaffolding has been elaborated in detail elsewhere [23,27–29] with theoretical and empirical relevance shown to both fraternal and egalitarian ETIs [29–31]. To briefly introduce the concept, I revisit two previously described examples—one relevant to fraternal transitions and the second to egalitarian transitions [28].

**Figure 1.**
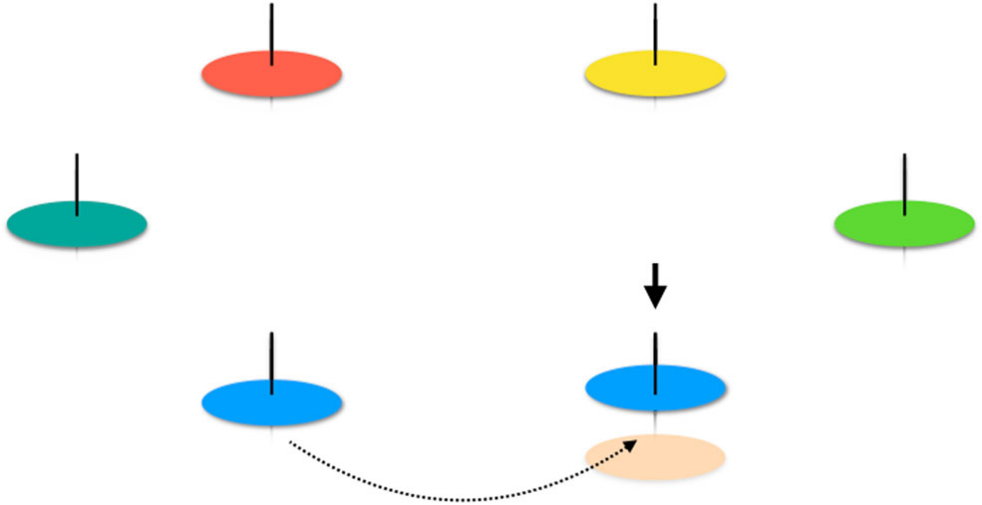
Ecological scaffolding: microbial mats acquire Darwinian properties. The cartoon shows six reeds in a pond. Surrounding each reed is a set of different microbial mat-forming types. Reeds are spaced so as to prevent confluent growth of mats, thus ensuring discreteness and thus mat-level variation. Consider that the orange mat occupying the reed marked with the solid arrow collapses and dies. Death provides opportunity for birth of a new mat, provided there exists a means of dispersal between reeds (by biotic or abiotic means). In this example, cells from the blue mat recolonize the vacant reed. The dispersal and recolonization event is akin to mat-level reproduction and, because the cells founding the new mat derive from the original mat, the offspring mat resembles the parental mat (there is heredity). Mats thus begin to take part in the process of evolution by natural selection by virtue of Darwinian properties that are exogenously determined. Additionally, selection acts over two time-scales: the doubling time of individual cells and the doubling time of mats—the latter directly tied to a mat-level death–birth process. Continued selection allows the possibility that Darwinian properties become endogenized, that is, they come to be determined by the activity of the collectives themselves with no need for an external scaffold. An early stage might be the evolution of a developmentally determined life cycle. Figure and caption adapted from [28].

With attention on fraternal transitions, for example, the evolution of multicellularity, Darwinian properties can be exogenously imposed on cells via an environment that affords nothing more than patchily distributed resources and the possibility of dispersal of cells between patches. Black *et al.* [23] provide a detailed theoretical account of a minimal ecological scaffold, but the concept is readily intuited from a plausible scenario drawn from nature (figure 1).

Consider a pond containing a genetically diverse population of unicellular types: cells are aerobic, planktonic and motile. Cellular metabolic activity reduces oxygen within the pond to levels that limit growth. Cells that reach the surface—where oxygen is abundant—stand to reap a significant growth advantage, but to do so they require physical structures upon which growth at the air–liquid interface can be anchored. It just so happens that this requirement is met by reeds distributed throughout the pond. Single colonizing cells adhere to reeds (one cell per reed) and give rise to cellular collectives (microbial mats). Mats are not buoyant and eventually perish owing to increasing mass. However, provided a mat can re-establish at the original reed, or around a new reed, via cells that disperse from an extant mat, then a process akin to collective-level reproduction occurs (figure 1).

In this example, cells have no innate capacity to become multicellular and no ‘intention’ of doing so. However, specific ecological circumstances cause cells to evolve as if members of multicellular collectives with Darwinian properties being exogenously imposed on cellular collectives: collective-level variation arises from placement of reeds (and capacity of cells to colonize reeds); periodic death of mats provides opportunity for extant mats to reproduce (reproduction is the consequence of dispersal). Because new mats are established from single cells, offspring mats resemble parental types.

The ensuing mat-level death–birth process, no matter how initially imperfect, causes mats to participate in the process of evolution by natural selection—as units in their own right. The collective-level process feeds back to affect the evolutionary dynamics of constituent cells, causing the fate of cells and collectives to become increasingly aligned [23]. After all, collectives composed of cells that produce a short-lived mat will themselves be short-lived, whereas cells that grow more slowly, perhaps a consequence of investment in production of adhesive polymers that enhance mat longevity, stand to prosper over the long term [24].

It further follows that continued evolution under such a scaffolded regime will deliver changes to collective-level function likely to enhance collective-level fitness. In particular, selection is expected to refine developmental processes underpinning evolution of a reproductive division of labour. This typically requires the evolution of life cycles such as those involving sequential alternations between soma-like mat cells and germ-like dispersing cells [30,32,33]. Precisely such innovations have been observed in laboratory-based experiments [30]. Further changes are likely to impact on the evolution of boundaries, discreteness and ability to distinguish self from other. Such modifications each amount to steps in a process by which exogenously imposed Darwinian properties evolve to become endogenous features of the nascent multicellular organism [34].

The second example—relevant to egalitarian transitions and inspired by concepts of the super-organism [35]—was first conceived as a thought experiment designed to be performed in a milli-fluidic device in which thousands of microbial communities could be propagated by serial transfer, while also being subject to a death–birth process based on some pre-determined community-level function [28,29].

**Figure 2.**
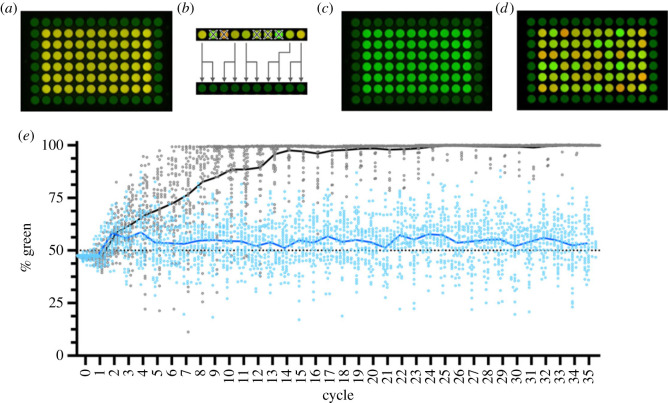
Ecological scaffolding: exogenous imposition of Darwinian properties on communities. (*a*) A fluorescent image of a 96-well microtitre plate 48 h after inoculation of the central 60 wells (containing rich medium) with an equal ratio of two isogenic genotypes of *Pseudomonas fluorescens* SBW25 that differ solely in the fluorescent protein each expresses (one GFP (green) and the other RFP (red), with an equal ratio of types generating communities that are yellow in colour). Confinement of communities to individual wells means that communities are discrete. Variation among communities arises from stochastic effects associated with sampling and is further influenced by differences in growth rate among red and green genotypes. Every 48 h, 30 communities are chosen, either at random, or on the basis of community colour, with two approximately 100-cell samples collected from each of the 30 chosen communities. The 30 non-selected communities are marked for extinction. Reproduction of communities involves transferring the two approximately 100-cell samples from each chosen community to two wells containing fresh medium in a new microtitre plate. (*b*) The death–birth process imposed on communities based on selection for yellowness (for clarity only a single row of wells is shown). Because offspring communities are derived from parental communities a degree of heredity is assured. However, in the absence of interactions, the phenotype of offspring colonies is subject to stochastic variation as a consequence of the sampling process. Nonetheless, communities are Darwinian, with collective-level Darwinian properties being exogenously imposed via the scaffolding strategy. As in figure 1, selection acts over two time-scales: the doubling time of individual cells and the doubling time of communities the latter directly tied to the community-level death–birth process. (*c*) The community-level phenotype after 35 reproductive (transfer) cycles in which the 30 communities chosen to reproduce are selected at random with no regard to community-level phenotype. As is evident, green has fixed in each instance. This is a consequence of a growth rate advantage to GFP-containing cells. (*d*) The community-level phenotype after 35 reproductive (transfer) events in which the 30 communities chosen to reproduce are selected based on community function (colour). Collective-level selection clearly counters cell-level selection. (*e*) The quantitative dynamics of community evolution over 35 cycles. Dots depict the colour of each community based on fluorescence signal imaged using a ChemiDoc MP Imaging System (BioRad). Grey dots (with line centred on the mean) show the trajectory for communities not subject to selection on colour. Blue dots (with line centred on the mean) depict the trajectory for communities subject to community-level selection. Continued community-level selection is predicted to drive the evolution of interactions that improve the parent–offspring relationship, thus endogenizing Darwinian properties and freeing communities from need for the externally imposed scaffold [29]. Dave Rogers and Ellen McConnell performed the experiment, analysed the data and produced the figures. Raw data are available in the electronic supplementary material.

A simple example involves a community of two bacterial cell types that differ solely in the colour of a fluorescent marker (one carrying red fluorescent protein (RFP, red) and the other green fluorescent protein (GFP, green)). Communities with an equal ratio of green to red—thus yellow—have by definition the highest fitness and hence are the most likely to persist. Experimental realization, via communities maintained in microtitre plates rather that a milli-fluidic device, is shown in figure 2.

Communities—comprising initially equal ratios of red and green cells—are packaged into the wells of microtitre plates. Such packaging ensures variation at the level of communities. Reproduction is effected by sampling cells from each community, with transfer of the sample to a new well replete with fresh medium. Heredity is delivered by the fact that offspring communities are established directly from a sample of cells derived from the parental community. Community-level selection unfolds via a birth–death process (figure 2*b*) that depends on community function (the colour yellow).

At periodic intervals the colour of each independent community is determined by placing the microtitre plate in a device capable of recording fluorescence. Half of the communities furthest from an equal ratio of red to green cells are marked for extinction. From the surviving communities (with colour closest to yellow), two samples of cells are taken and transferred to two wells in a fresh plate: each viable community thus leaves two offspring communities. In order to understand the effect of community-level selection, a control is included in which communities marked for death and birth are chosen at random and with no regard for community-level colour.

In the absence of community-level selection, the fate of communities is driven by cell-level fitness. Green cells have a growth rate advantage over red cells and thus green rapidly fixes at the community level. In marked contrast, selection on communities, based on a community-level function (colour), counters cell-level fitness effects and allows maintenance of yellow communities (figure 2*e*).

Although there are genealogical connections between parent and offspring communities, it is important to recognize that the likelihood that offspring communities resemble parental communities—at least at the start of the experiment—is low. This is because there is no pre-existing interaction between red and green cells. As a consequence, heredity is determined solely by vagaries arising from stochastic effects associated with sampling a small number of cells from a large community. As is evident in figure 2, community-level selection readily counters these stochastic effects, precisely as envisaged by the stochastic corrector model [36]. Continued community-level selection is expected to favour the spread of innovations that result in increasing alignment of the reproductive fates of red and green cells, and thus drive the evolution of heredity [29].

For intuitive understanding, consider that at the start of the experiment just one in ten communities bears close resemblance to the phenotype of parental communities, with those offspring communities whose colour is significantly worse facing elimination at the next round of selection. Imagine, however, that within one community a mutant red cell arises with increased propensity to interact with green cells, such that half of all offspring communities now closely resemble the parental type. Clearly, a community leaving offspring communities that with high probability inherit the parental phenotype will rapidly replace communities whose offspring show a low fidelity of inheritance.

Innovations that underpin the evolution of heredity are interactions. Drawing from recognized egalitarian transitions, extreme scenarios might involve acquisition of the gene encoding RFP by the green bacterium (or *vice versa*), or engulfment of the red bacterium by the green (or *vice versa*). Less radical solutions stand to arise from physical attachment of red and green cells, or from the evolution of interactions mediated through allelopathic or density-dependent effects.

The latter receives strong support from theoretical studies where models that allow evolutionary change in parameters that affect density-dependent interactions show the emergence of developmental-like processes that correct for stochastic fluctuations in the ratio of founding cells [29]. In fact, so effective is the ‘developmental corrector’ that the parent–offspring relationship continues to remain heritable even after removal of exogenously imposed scaffolds. As in the reed example above, changes such as these mark steps toward the endogenization of Darwinian properties (at the collective level).

## 4. Plausible evolutionary transitions in individuality in humans

The three intertwined Darwinian properties of variation, differential fitness and heredity are each well defined, but explanations for their origin differs depending on the nature of the ETI. For fraternal transitions, the primary challenge concerns explanations for collective-level reproduction. Because cells of the collective are essentially identical, the nature of heredity follows from understanding mode of reproduction.

For egalitarian transitions, the principal question concerns the origin of heredity. Because lower-level entities are distinct, and each endowed with replicative capacity, the nature of collective-level heredity follows from understanding the causes of alignment of replicative fates. In essence, this involves understanding the bases of interactions that cement partner alliances such that the two lower-level entities come to replicate as one. For example, chromosomes arose from interactions that placed once independently replicating genes on a single self-replicating molecule [37]; the eubacterium–REPIN symbiosis arose from previously autonomous transposons becoming trapped within the eubacterial genome [38]. For the eukaryotic cell, engulfment (or invasion) of one prokaryotic cell by the other sealed the replicative fates of both partners [39]. Turning to consider plausible future ETIs in humans wrought by the possibilities presented by the exogenous imposition of Darwinian properties, it is important to recognize these distinctly different challenges.

In the examples of ecological scaffolding presented above, attention was restricted to abiotically determined features of the environment, such as patchily distributed resources (to discretize collectives and fuel growth of cells) and a means of dispersal (to effect movement of cells between patches). As I move to consider opportunities for scaffolding in human populations, behavioural, cultural, political, religious and legal structures greatly extend prospects for external imposition of Darwinian properties [40,41]. Such structures might constitute societal norms with adherence ensured by way of social sanctions [42], or they may be imposed via legal structures and enforced by state-level policing [43].

Routes by which humans might undergo future fraternal ETIs are in principle conceivable, but in reality, they are likely unachievable and sit largely within the realms of science fiction. Humans exist within societies and societies differ profoundly one to another. Variation thus exists at the level of societies. Even though human societies share many features with social insects, human societies do not leave offspring societies that resemble parental societies in the way that beehives leave offspring colonies [44].

Selection may nonetheless operate on societies [45,46], such that one society may expand and replace another, but this is largely an ecological process akin to replacement of red squirrels by grey squirrels in parts of the United Kingdom. Political, cultural and religious ideas may similarly spread via horizontal transmission; thus selection may operate on properties of societies [40], generating group-level adaptations [47,48], but again, there is little sense of societies acting as units of selection in their own right, as for example, is the case with bee colonies.

Drawing upon ideas from ecological scaffolding, and leaving aside ethical concerns, it would be possible, in principle, to impose, via an overarching politically or religiously driven structure, rules that assign people to groups, restrict reproduction so that it occurs exclusively among members of each group, and forbid migration between groups. Such an arrangement would ensure variation at the level of groups. But what of reproduction and heredity?

Consider—again, ethical concerns aside—a politically imposed legal structure that enforces fragmentation of groups into two (or more) upon realization of some exogenously imposed group-level function. Such a function may be simply a product of group size, but could also be dependent on achievement of some group-level property. According to externally imposed rules, those groups achieving the necessary threshold for fragmentation would replace those groups that had failed to achieve this functionality. A birth–death process is thus effected at the group-level of groups.

From a purely genetic perspective, groups are chimeras defined by individual humans, with offspring groups being similarly chimeric. There is thus no guarantee that offspring groups will resemble parental groups—even though genealogical connections exist between parent and offspring groups [9]. Nonetheless, with such an arrangement, and with sufficiently strict and enforced legal structures, groups will participate in the process of evolution by *artificial* selection, with group-level heredity being assured by continual enforcement of legal structures. Of course, such an arrangement pre-supposes the existence of some overall organizational system that continually imposes rules from outside the groups themselves. Just how this might be sustained is difficult to envisage.

Again, drawing from concepts of ecological scaffolding, in which Darwinian properties that are externally imposed evolve to become endogenous properties of the evolving collectives, it is pertinent to ask whether endogenization might ever occur. I think possibilities for endogenization are negligible. Given genetic heterogeneity of groups, the only possibility for ensuring group-level heredity in the absence of externally imposed and policed rules would be emergence of a set of cultural norms—intrinsic to each group—to which all individuals voluntarily adhere. Given the potency of selection on individuals to defect from norms that are likely costly to individual humans, it is inconceivable that groups will ever participate as units of selection in their own right in the absence of draconian rules that are externally imposed.

But there is one collective-level innovation that would make a difference, and that is if groups somehow evolved a reproductive division of labour akin to that found in eusocial societies. If achieved—which seems an infinitely remote possibility—groups would be defined by a single reproductive lineage (the equivalent of a queen bee), offspring within groups would be kin, conflicts among kin would thus be minimal, and non-reproductive humans would assume the role of soma, serving solely as vehicles for the germline (the queen). Moreover, offspring groups would show high similarity to parental groups. Such groups would manifest paradigmatic Darwinian properties and have no choice but to participate in the process of evolution by natural selection as units in their own right [44]. Continued group-level selection would drive ‘de-Darwinization’ of individual humans, enforcing their integration as components of a corporate collective replete with developmental control and mechanisms of self-policy [12]. The reality of such an ETI would be truly horrific when viewed from the stance of currently extant humans.

Turning attention to scenarios for egalitarian transitions in individuality, I see far greater potential for ecological scaffolding to effect human ETIs. The range of potential partners is considerable, ranging from microbes that compose the microbiome, through to interactions with AI. Recognition that humans and their microbiomes might constitute units of selection has received much attention [49–51] and I will not further consider this. Suffice to say that if the microbiome was strictly vertically inherited from the mother to baby during birth, and there was no migration thereafter, then humans together with their microbiomes would evolve as units of selection in their own right—precisely as evident in insects that harbour obligate, vertically transmitted, endosymbionts [49]. It is conceivable that medical practices could be implemented that increased fidelity of microbiome inheritance, thus ensuring paradigmatic manifestation of heritable variance in fitness at the level of humans and associated microbiomes.

Associations between humans and AI devices are now common place. Many humans own and carry a mobile phone. Information provided via the computational power of mobile devices affects how we function. Even in the absence of sophisticated machine learning algorithms, applications—and the algorithms they encode—influence information received and thus affect world views, alter states of mind, play roles in health and disease prevention, underpin partner choice, determine particulars of travel, and impel purchase decisions. In short, interactions with mobile devices already have fitness-affecting consequences, but with advances in AI, and especially algorithms capable of learning from—and responding to—information received from individual (human) users, interactions between humans and AI devices stand to be reactive to changing circumstances, with far-reaching effects on fitness.

**Figure 3.**
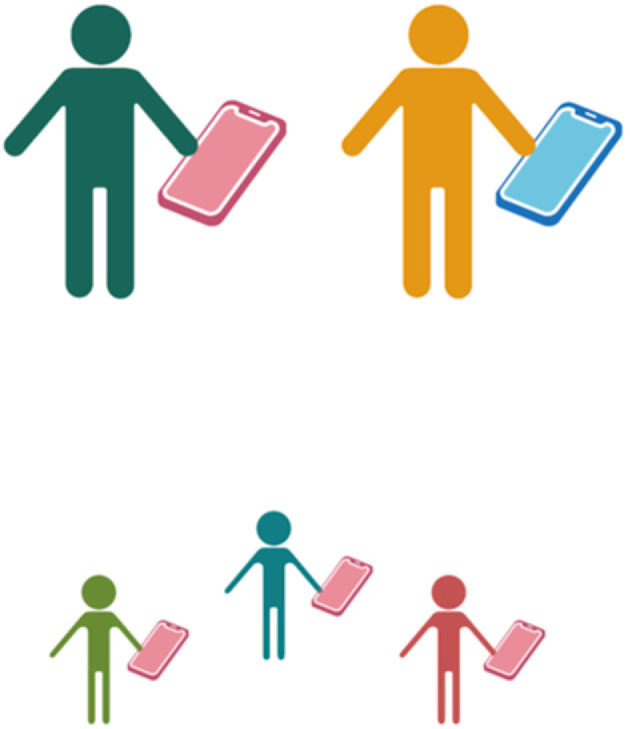
Exogenous imposition of Darwinian properties on individual humans and personal AI devices. Humans are Darwinian: humans vary one to another, they reproduce, and offspring resemble parental types, but machine learning algorithms on AI devices are not Darwinian. While algorithms on individual devices vary (a consequence of feedback between individual humans and the machine learning algorithms on individual devices), AI devices do not reproduce. Nonetheless, AI devices can reproduce via a simple human intervention, which ensures that information stored on parental devices is copied to fresh devices given to offspring. To effect an evolutionary transition in individuality between humans and AI devices all that is required are rules that make it obligatory for humans to pass the contents of their AI devices to fresh devices provided to offspring. The information could be passed from a single parent (as indicated by the pink devices here) or inheritance could be bi parental or via some other combination of means. As a consequence of this societally imposed scaffold, the collective—individual humans plus their individual AI devices—become a single higher-level entity that participates directly in the process of evolution by natural selection. Figure produced using BioRender.

It is a relatively trivial matter to ensure that humans and their AI devices acquire collective-level Darwinian cohesiveness. Humans are Darwinian entities. AI devices are not, but there is no requirement for AI devices to be somehow engineered into the germline to ensure that AI devices plus their human counterparts—together—become Darwinian units in their own right. All that is needed is a process by which human offspring receive an AI device onto which algorithms shaped by parental interactions with AI devices are copied (figure 3). To some extent, this already happens. Even if not intentional, mobile devices are often passed from parent to child replete with parental applications and associated information. Even in cases where a child receives a new device, it is likely that applications and subscriptions from parents are transmitted to offspring. In effect, this is cultural transmission, albeit via a device rather than by direct learning from parents.

But consider the possibility that a societal construct imposes a legal framework that requires every human to have an AI device, and for the contents of that device to be transferred to a device provided to offspring. Adherence to this simple instruction means that humans and AI devices become units of selection. In principle, there is little difference from early stages in evolution of the eukaryotic cell. However, absent at the outset is full dependency of the human on its AI device (although the reverse is true from the start), but continued selection on the partnership stands to rapidly effect such dependency. Indeed, the ‘green–red’ community-level selection experiment outlined above (figure 2), with demonstration from theory of the relative ease by which interactions between partners stand to evolve [29], shows what is to be expected even in the absence of systems responsive to opportunities from alterations in behaviour.

Recognition that ETIs of an egalitarian kind, between humans and AI devices, could be effected so readily brings considerable intrigue, but also raises numerous issues and concerns surrounding the transmission of information. Should information be passed from one parent, or both parents, and if from one parent, should transmission be tied to gender [52]? Lessons from transmission of mitochondria indicate that transmission from a single parent is likely to prove most potent. Indeed, were mitochondria biparentally inherited, conflicts detrimental to offspring are likely to arise, with no way to prevent the spread of deleterious mitochondrial mutations [53].

Of significant concern are the myriad opportunities for intervention of the state—or cultural/religious groups—in the process of information transmission. Even if there were ways by which such intervention could be avoided, it is difficult to see, given the current state of AI and machine learning, that information transmitted could ever be free of external influences. Worst case scenarios might involve malevolent intervention such that algorithms that bias behaviour toward goals that further the interests of particular political, cultural or religious groups are enforced.

In suggesting a plausible route for evolutionary transitions between humans and AI devices, particular importance has been placed on AI. Some might reasonably point to the fact that AI devices are no different from other kinds of technical objects [54–56], and thus arguments that AI should be given special significance are misplaced, or at least the scenario outlined above should be extended to encompass other kinds of objects.

Humans have long-crafted tools: instruments for hitting, such as hammers, being prime examples. Like AI, hammers can be viewed as technical extensions of the mind [56]. Their form and function stand, under certain circumstances, to affect human fitness. Moreover, the design of hammers evolves as a consequence of feedback among a range of factors, including function, aesthetics, economics and utility—at the level of both individual humans (the constructors) and groups of humans (the users and beneficiaries)—with design transmitted via various mechanisms of cultural exchange. As with AI, interactions among humans and hammers will evolve, effecting ever greater co-dependency between human and object.

From this perspective, indeed, the interactions between humans and hammers, and humans and AI, are strikingly similar. Arguably, interactions between humans and both kinds of object could be seen as having similar significance, and both are worthy of consideration from the view point of egalitarian transitions in individuality, along with similar possibilities for scaffolding Darwinian properties on combinations of humans and objects, in general. But there is an important distinction that stems from differences in capacities of hammers and AI to learn and respond, in real time, to human action. Hammers manifest such ability in the most limited sense and only in terms of the relationship between mind and object. AI, however, is endowed with extensive, largely independent, capacity to learn and respond, and provoke immediate actions on the part of affected humans. With this comes opportunity for rapid coevolutionary dependency leading to fitness alignment of human and AI. Indeed, ever-increasing development of sophisticated algorithms is likely to make future AI devices ever more potent, with such algorithms and linked devices even determining, through largely independent action, modes of information transmission.

## 5. Conclusion

On first thought, the possibility that humans might undergo some future ETI seems to fall within the bounds of science fiction. However, as I have argued here, egalitarian ETIs involving humans and AI devices are imminently realizable. Awareness stems, firstly, from realization that ETIs only occur when higher-level collectives acquire properties that allow participation in the process of evolution by natural selection, and secondly, from understanding that collective-level Darwinian properties require evolutionary explanation. One kind of explanation that avoids the mistake of pre-supposing the presence of Darwinian properties at the level at which they emerge recognizes that heritable variance in fitness at higher organizational levels can be exogenously determined by circumstances that are external to the evolving entities.

In adopting an externalist viewpoint, and turning attention to possible human ETIs, it becomes apparent that the raw material necessary to effect egalitarian ETIs between humans and AI devices is close at hand. Required is nothing more than fitness-affecting interactions between humans and AI devices that continually evolve in response to information each receives from the other, combined with a means of ensuring that such interactions are passed to offspring. The latter is achieved simply via changes in behaviour that ensure that when humans reproduce, the contents of parental AI devices are copied to AI devices inherited by offspring. Co-evolution between the two partners will lead to increasing dependency of humans on AI devices and thus externally imposed Darwinian properties are expected to rapidly evolve to become endogenous features of the new organizational level.

## Data Availability

The data are provided in the electronic supplementary material [57].
